# Influenza and Current Guidelines for its Control

**DOI:** 10.1155/S106474490100031X

**Published:** 2001

**Authors:** Stanley A. Gall

**Affiliations:** 1 Department of Obstetrics and Gynecology University of Louisville 550 South Jackson Street Louisville KY 40202 USA


*Infect Dis Obstet Gynecol 2001;9:193–195*

### Influenza and current guidelines for its control

**Stanley A. Gall**

*Department of Obstetrics and Gynecology, University of Louisville School of Medicine, Louisville, KY*

Influenza epidemics occur during the winter months in the Northern Hemisphere (October–March) and cause approximately 20 000 deaths per year in the US[1]. Influenza viruses cause disease at all ages with infection rates highest among children, especially those 0–6 months of age, and serious illness and death rates highest among those aged 65 years and over. Persons at any age who have medical conditions that place them at high risk for complications from influenza have increased morbidity and mortality[2].

Annual influenza vaccination is the primary method for preventing influenza and its sequelae. The primary target groups recommended for annual vaccination include:

1) all persons aged 50 or over;
2) persons of any age who have chronic medical conditions;
3) persons who live with or care for persons at high risk, i.e. health care workers; and
4) all women who are pregnant during the influenza season (October–March).

Vaccination is associated with a reduction in: influenza-related respiratory illness and physician visits among all age groups; hospitalization; death among children with persistent high-risk otitis media; and work absenteeism among adults[3–5].

Epidemics of human influenza are caused by two types: influenza A and influenza B. Influenza A viruses are characterized into subtypes on the basis of their two surface antigens: hemagglutinin (H) and neuraminidase (N). Influenza B viruses are not subtyped. Since 1977, Influenza A ($H_1N_1$) viruses, Influenza A ($H_3N_2$) viruses, and Influenza B viruses have been in worldwide circulation. New variants arise due to point mutations that occur during viral replication (antigenic drift). The development of antigenic variance through antigenic drift is the virologic basis for seasonal epidemics and the reason for incorporation of one or more new strains in each year's vaccine.

The risk of serious complications, hospitalizations and mortality from influenza is higher among persons aged 65 years and over, very young children aged 0–6 months, and persons of any age with high-risk medical conditions[1]. Rates of influenza-associated hospitalizations have varied substantially by age group (Table 1). Children aged 0–4 years have hospitalization rates approximately 500/100 000 population for those in high-risk groups to 100/100 000 hospitalizations for those without high-risk conditions. The information in Table 1 shows the 0–11 month age group with the low estimate for children age 6–11 months and the high estimate for children aged 0–5 months. Few studies are available to assess the impact of maternal immunization with influenza vaccine in the neonate. England and colleagues have championed the concept of maternal immunization to protect both the mother and neonate in months 0–6 of life[9,10]. Since infants aged 0–6 months have a risk of hospitalization as great as or greater than persons 65 years of age or over, the only way to protect pregnant women and their offspring is to administer the vaccine in the second or third trimester of pregnancy. Vaccination would be required during pregnancy to allow for optimal antibody levels in the infant.

Correspondence to: Stanley A. Gall, Department of Obstetrics and Gynecology, University of Louisville, 550 South Jackson Street, Louisville, KY 40202, USA. Email: sagall@louisville.edu

**Table 1** Estimated rates of influenza-associated hospitalization by age group

| *Study years* | *Age group* | *Hospitalizations/100,000 persons at high risk* | *Hospitalizations/100,000 persons not at high risk* |
|---|---|---|---|
| 1973–1993[6] | 0–11 m | 1900 | 496–1038 |
| | 1–2 y | 800 | 186 |
| | 3–4 y | 320 | 86 |
| | 5–14 y | 92 | 41 |
| 1992–1997[7] | 0–23 m | | 144–187 |
| | 2–4 y | | 0–25 |
| | 5–17 y | | 8–12 |
| 1968–1969[8] | 15–44 y | 56–110 | 23–25 |
| | 45–64 y | 392–635 | 13–23 |
| | ≥ 65 y | 399–518 | — |
| 1968–1995[8] | < 65 y | | 20–42 |
| | ≥ 65 y | | 125–228 |

Vaccination levels in persons aged 65 or over increased from 33% in 1989 to 63% in 1997 and 1998[3]. Limited information is available regarding the use of influenza vaccine in pregnant women. Women aged 18–44 without diabetes reported in 1999 that during pregnancy they were less likely to receive an influenza vaccination than when not pregnant (9.6% compared with 15.7%)[3]. This indicates low compliance with ACIP's recommendations for pregnant women. In a subsequent study[11], influenza vaccine acceptance by pregnant women occurred in 71% of those who were offered the vaccine. Gonik and colleagues[12] surveyed obstetrician gynecologists in Michigan and determined that only 39% gave influenza vaccine to obstetrics patients, but 86% agreed that pregnant women's risk for influenza-related morbidity and mortality increased in the second and third trimesters of pregnancy. Targeted groups for vaccination include:

1) all persons aged 50 years and over;
2) women who will be pregnant during the influenza season;
3) residents of nursing homes and chronic care facilities of any age;
4) adults and children who have chronic disorders of the pulmonary or cardiovascular systems including asthma and smoking;
5) adults and children who require regular medical examination or who have been hospitalized in the last year because of diabetes mellitus, renal or hemalologic disease, or immunosuppression; and
6) health care providers.

The impact of influenza during pregnancy can be devastating. Influenza-associated excess deaths were seen in the pandemics of 1918–19 and 1957–58[13]. Case-facility ratio reached 60% in the 1918–19 pandemic. Alterations of pregnancy physiology are detrimental to, and increase severity of, all types of pneumonias in pregnancy. There are increases in heart rate, stroke volume and oxygen consumption, decreases in lung capacity, and changes in immunologic function[14]. Neuzil and colleagues reported on the impact of influenza during 17 interpandemic influenza seasons, and found the relative risk for hospitalization with cardiorespiratory conditions increased from 1.4 during weeks 14–20 of pregnancy to 4.7 during weeks 37–42, compared with women who were 1–6 months postpartum[15].

The current trivalent vaccine is an inactivated vaccine and is considered safe in all trimesters of pregnancy. A study of influenza vaccination of over 2000 pregnant women[16] demonstrated no adverse fetal effects associated with the vaccine. Influenza vaccine administration in the first trimester, while safe, may occur with a coincidental spontaneous abortion and consideration should be given to defer vaccine until the second trimester. Influenza vaccine does not affect the safety of

**Table 2** Month of peak influenza activity during 19 influenza seasons[3]

| | *December* | *January* | *February* | *March* |
|---|---|---|---|---|
| Number of years with peak influenza activity | 4 (21%) | 5 (26%) | 7 (37%) | 3 (16%) |

mothers who are breastfeeding, or their infants – breastfeeding is not a contraindication for vaccination.

Influenza vaccinations should be administered during the months of October–March. Table 2 shows the month of peak influenza activity during 19 influenza seasons in the US. The implication is that the influenza season is not over by November but pregnant women should be vaccinated up to and including March.

Influenza vaccine should be given intramuscularly (IM) with a needle ≥ 1 inch in the deltoid muscle. Inactivated vaccine should not be given to persons who have anaphylactic hypersensitivity to eggs or to other components of the influenza vaccine.

Influenza can cause serious complications and mortality. Pregnant women of all ages are a targeted group as are all people aged 50 or over. Influenza vaccine reduces significant morbidity and there is preliminary data suggesting a reduction of influenza and otitis media in infants aged 0–6 months.

### REFERENCES

1. Luis KJ, Kendal AP. Impact of influenza epidemic on mortality in the United States for October 1972–May 1982. *Am J Public Health* 1987;77: 712–16
2. Barker, WH. Excess pneumonia and influenza associated hospitalizations during influenza epidemics in the United States 1970–1978. *Am J Public Health* 1986;76:761–5
3. Prevention and control of influenza. Recommendations of the Advisory Committee on Immunization Practices (ACIP). *MNWR* 2001;50(RR-4): 1–45
4. Nicol KL, Lind A, Margolis KL, *et al.* Effectiveness of vaccination against influenza in healthy working adults. *N Engl J Med* 1995;333:889–93
5. Clements DA, Langdon L, Bland C, Walter, E. Influenza A vaccine decreases the incidence of otitis media in 6 to 30 month old children. *Arch Pediatr Adolesc Med* 1995;149:1113–7
6. Neuzil KM, Wright PF, Mitchell EF, *et al.* Effect of influenza on hospitalizations, outpatient visits, and courses of antibiotics in children. *N Engl J Med* 2000;342:225–31
7. Barker WH, Mullooly JP. Impact of epidemic type A influenza in a defined population. *Am J Epidemiol* 1980;112:798–811
8. Simonsen L, Fukuda K, Schonberger LB, Cox W. Impact of influenza epidemics on hospitalization. *J Infect Dis* 2000;181:831–7
9. Glezen WP. Serious morbidity and mortality associated with influenza epidemics [Review]. *Epidemiol Rev* 1982;4:25–44
10. Englund JA, Mbawuike IN, Hammill H, *et al.* Maternal immunization with influenza or tetanus toxoid for passive antibody protection in young infants. *J Infect Dis* 1993;168:647–56
11. Yeager DP, Toy EC, Baker B III. Influenza vaccination in pregnancy. *Am J Perinatal* 1999;16:283–6
12. Gonik B, Jones T, Contrearas D, *et al.* Obstetrician-gynecologists role in vaccine preventable disease and immunization. *Obstet Gynecol* 2000;96:81–4
13. Widelock D, Csizmas L, Klein S. Influenza pregnancy and fetal outcome. *Public Health Rep* 1963; 78:1–11
14. Shahab SZ, Glezen WP. Influenza virus. In Gonik B, ed. *Viral diseases in pregnancy*. New York: Springler-Verlag, 1994:215–23
15. Neuzil KM, Reed GW, Mitchell EF, *et al.* Impact of influenza on acute cardio-pulmonary hospitalizations in pregnant women. *Am J Epidemiol* 1998; 148:1094–102
16. Heinonen OP, Shapero S, Monsen RR. Immunization during pregnancy against poliomyelitis and influenza in relationship to childhood malignancy. *Int J Epidemiol* 1973;2:229–35

Received 10/02/01; Accepted 10/03/01
